# Supplementary material for: Characteristics and Outcomes of Patients with Delftia acidovorans Infections: a Retrospective Cohort Study
Source: Microbiol Spectr. 2022 Jul 14;10(4):e00326-22. doi: 10.1128/spectrum.00326-22 (PMC9431703; doi:10.1128/spectrum.00326-22)
Supplement: Supplemental file 1 — Tables S1 and S2. Download spectrum.00326-22-s0001.pdf, PDF file, 0.1 MB [file spectrum.00326-22-s0001.pdf]

Supplementary Table 1: Comorbidities identified at the time of *D. acidovorans* infection.

**Comorbidities** (alphabetically)	number
**Bone marrow transplant recipient**	<3
**Cardiovascular disease**	
Myocardial infarction	3
Congestive heart failure	4
Peripheral vascular disease	5
**Cerebral vascular disease**	7
**Chronic obstructive pulmonary disease**	4
**Congenital syndromes**	5
**Connective tissue disease**	3
**Cystic fibrosis**	4
**Diabetes**	3
**Hematologic malignancies**	15
**Interstitial lung disease**	<3
**Liver disease**	<3
**Neuromuscular disease**	<3
**No known comorbidity**	<3
**Primary ciliary dyskinesia**	6
**Renal dysfunction**	4
**Solid cancer**	10
**Solid organ transplant recipient**	<3

Supplementary Table 2: Number of patients who received antibiotic within before or after the first positive *D. acidovorans.*

Antibiotic	Number of patients who received antibiotic within 3 months before the first positive *D. acidovorans*	Number of patients who received a new antibiotic after the first positive *D. acidovorans**
Penicillin or Ampicillin	3	<3
Amoxicillin	6	3
Amoxicillin / Clavulanic acid	<3	-
Dicloxacillin	3	-
Pivmecillinam	<3	-
Piperacillin/Tazobactam	3	3
Cefuroxime	<3	<3
Doxycycline	<3	-
Linezolid	<3	-
Vancomycin	3	<3
Clarithrmoycin or Azithromycin	6	-
Trimethoprim / Sulfamethoxazole	3	<3
Moxifloxacin or Ciprofloxacin	10	9
Nitrofurantoin	<3	-
Meropenem	4	8
Gentamicin or Tobramycin	4	-
Colistin	4	<3
Rifampicin	<3	-
Metronidazol	<3	-

*If the antibiotic was started before the positive *D. acidovorans* was not counted here.
- No information was available.
